# Prognostic value of *KRAS *genotype in metastatic colorectal cancer (MCRC) patients treated with intensive triplet chemotherapy plus bevacizumab (FIr-B/FOx) according to extension of metastatic disease

**DOI:** 10.1186/1741-7015-10-135

**Published:** 2012-11-08

**Authors:** Gemma Bruera, Katia Cannita, Daniela Di Giacomo, Aude Lamy, Giancarlo Troncone, Antonella Dal Mas, Gino Coletti, Thierry Frébourg, Jean Christophe Sabourin, Mario Tosi, Corrado Ficorella, Enrico Ricevuto

**Affiliations:** 1 Medical Oncology, S. Salvatore Hospital, University of L'Aquila, L'Aquila, Italy; 2 Department of Experimental Medicine, University of L'Aquila, L'Aquila, Italy; 3 Laboratory of Tumor Genetics, University Hospital, Rouen, France; 4 Department of Biomorphologic and Functional Sciences, University Federico II, Napoli, Italy; 5 Pathology Department, S. Salvatore Hospital, L'Aquila, Italy; 6 INSERM U614, University of Rouen, Rouen, France; 7 Department of Pathology, INSERM U614, Rouen University Hospital, Rouen, France

**Keywords:** disease extension, intensive regimen, *KRAS *mutations, metastatic colorectal cancer, triplet chemotherapy plus bevacizumab

## Abstract

**Background:**

Bevacizumab (BEV) plus triplet chemotherapy can increase efficacy of first-line treatment of metastatic colorectal cancer (MCRC), particularly integrated with secondary liver surgery in liver-limited (L-L) patients. The prognostic value of the *KRAS *genotype in L-L and other or multiple metastatic (O/MM) MCRC patients treated with the FIr-B/FOx regimen was retrospectively evaluated.

**Methods:**

Tumoral and metastatic samples were screened for *KRAS *codon 12 and 13 and *BRAF *mutations by SNaPshot and/or direct sequencing. Fit MCRC patients <75 years were consecutively treated with FIr-B/FOx regimen: weekly 12-h timed flat-infusion/5-fluorouracil (TFI 5-FU) 900 mg/m^2^, days 1, 2, 8, 9, 15, 16, 22 and 23; irinotecan (CPT-11) 160 mg/m^2 ^plus BEV 5 mg/kg, days 1, 15; oxaliplatin (OXP) 80 mg/m^2^, days 8, 22; every 4 weeks. MCRC patients were classified as L-L and O/MM. Activity and efficacy were evaluated and compared using log-rank test.

**Results:**

In all, 59 patients were evaluated: 31 *KRAS *wild-type (53%), 28 *KRAS *mutant (47%). At 21.5 months median follow-up, objective response rate (ORR), progression-free survival (PFS) and overall survival (OS) were, respectively: *KRAS *wild-type 90%, 14 months, 38 months; *KRAS *mutant 67%, 11 months, 20 months. PFS and OS were not significantly different. PFS and OS were significantly different in L-L compared to O/MM evaluable patients. In *KRAS *wild-type patients, clinical outcome of 12 L-L compared to 18 O/MM was significantly different: PFS 21 versus 12 months and OS 47 versus 28 months, respectively. In *KRAS *mutant patients, the clinical outcome of 13 L-L compared to 14 O/MM was not significantly different: PFS 11 months equivalently and OS 39 versus 19 months, respectively.

**Conclusions:**

The *KRAS *genotype wild-type and mutant does not significantly affect different clinical outcomes for MCRC patients treated with the first-line FIr-B/FOx intensive regimen. *KRAS *wild-type patients with L-L disease may achieve a significantly prolonged clinical outcome due to integration with secondary liver surgery, with respect to *KRAS *mutant patients.

## Background

Triplet regimens consisting of chemotherapeutic drugs, or doublets plus bevacizumab (BEV) (anti-vascular endothelial growth factor monoclonal antibody) or cetuximab (anti-epithelial growth factor receptor (EGFR) monoclonal antibody) in EGFR-overexpressing and *KRAS *wild-type metastatic colorectal cancer (MCRC), reported overlapping activity and efficacy in phase III trials, ranging between objective response rate (ORR) 39% to 68%, progression-free survival (PFS) 7.2 to 10.6 months, overall survival (OS) 19.9 to 26.1 months [1]. In 'fit' MCRC patients, these first-line options, integrated with secondary resection of liver metastases, significantly increased survival over doublet regimens [1,2]. More intensive medical treatment consisting of triplet chemotherapy plus targeted agents can further increase activity, thus raising resection rate of liver metastases and clinical outcome [1-5]. Phase II studies, by Masi *et al. *[3], and by our group [4], proposed BEV addition to triplet chemotherapy, according to FOLFOXIRI/BEV or FIr-B/FOx schedules, reaching ORR 77% and 82%, median PFS 13.1 and 12 months, median OS 30.9 and 28 months, as first-line treatment of MCRC patients. Liver metastasectomies were performed in 32% and 26% overall and 40% and 54% liver-only patients, respectively. Thus, MCRC patients with liver-limited (L-L) disease, integrating FIr-B/FOx intensive regimen and secondary liver surgery significantly improved clinical outcome compared to MCRC patients with multiple metastatic disease, up to median PFS 17 months and median OS 44 months [6].

Gain-of-function mutations of *RAS, BRAF, PIK3CA *genes, or loss of tumor suppressor function of *PTEN*, resulting in continuous activation of the RAS-mitogen-activated protein kinase (MAPK) or phosphoinositide 3-kinase (PI3K) pathways, characterize most colorectal cancers (CRC) [7-9]. *KRAS *mutations represent an early event in colorectal tumorigenesis [10,11] and occur in 35% to 45% of CRC, mostly represented by codon 12 c.35 G>A (32.5%) [12,13], c.35 G>T (22.5%) [11,12], and codon 13, prevalently c.38 G>A, transversions [14]. They impair intrinsic GTPase activity of KRAS, and lead to constitutive, growth factor receptor-independent activation of downstream signaling [15]. *BRAF *mutations, prevalently c.1799 T>A (V600E) mutation, characterize 4.7% to 8.7% of CRC [16-20].

Clinical outcome (PFS, OS) according to wild-type and mutant genotype assesses the prognostic relevance of a specific biomarker, potentially including the predictive role of effectiveness of treatment strategies. In randomized studies, the predictive relevance of wild-type or mutant genotype can also be specifically assessed by comparing experimental and control arms. The reported median OS values of *KRAS *wild-type and mutant MCRC patients treated with irinotecan, 5-fluorouracil and leucovorin (IFL) plus BEV were 27.7 and 19.9 months, respectively [18,21]. The prognostic relevance of *KRAS *or *BRAF *wild-type compared to *KRAS *or *BRAF *mutant genotype was not significantly different, even though the hazard ratio (HR) was 0.64 and 0.38, respectively. A significantly better prognosis was reported only when *KRAS/BRAF *wild-type patients were compared with patients harboring mutations in the *KRAS *or *BRAF *genes (HR 0.51) [18]. *KRAS *wild-type genotype significantly predicts a favorable clinical outcome of anti-EGFR or anti-vascular endothelial growth factor (VEGF) drugs added to doublet chemotherapy [18,21-23]. In *the KRAS *mutant genotype, BEV addition to IFL significantly prolonged PFS up to 9.3 months, without increasing OS and activity, compared to IFL [18,21].

Here, we report a retrospective exploratory analysis evaluating the prognostic value of the *KRAS *genotype in MCRC patients enrolled in a previously reported phase II study [4] and in an expanded clinical program proposing FIr-B/FOx intensive regimen as first-line treatment, also verifying recently reported significantly greater effectiveness in L-L compared to other or multiple metastatic (O/MM) patients [6].

## Methods

### Patient eligibility

MCRC patients were enrolled in a previously reported phase II study [4] and in the expanded clinical program proposing FIr-B/FOx association as first-line treatment. Patients were eligible if they had a histologically confirmed diagnosis of measurable MCRC; were age 18 to 75 years; had World Health Organization (WHO) performance status ≤2; had adequate hematological, renal and hepatic functions; and had a life expectancy more than 3 months. The study was approved by the Local Ethical Committee (Comitato Etico, Azienda Sanitaria Locale n.4 L'Aquila, Regione Abruzzo, Italia) and conducted in accordance with the Declaration of Helsinki. All patients provided written, informed consent.

### Schedule

The FIr-B/FOx regimen was developed from previously reported doublet and triplet chemotherapy schedules [24,25], consisting of weekly timed flat-infusion/5-fluorouracil (TFI 5-FU), without leucovorin, associated to weekly alternating irinotecan (CPT-11)/BEV or L-oxaliplatin (OXP) [4]: TFI 5-FU (Fluorouracil Teva; Teva Italia, Milan, Italy), 900 mg/m^2^/day, over 12 h (from 10:00 pm to 10:00 am), days 1, 2, 8, 9, 15, 16, 22 and 23; CPT-11 (Campto; Pfizer, Latina, Italy), 160 mg/m^2^, days 1, 15; BEV (Avastin; Roche, Welwyn Garden City, United Kingdom), 5 mg/kg, days 1, 15; l-OXP (Eloxatin; Sanofi-Aventis, Milan, Italy), 80 mg/m^2^, days 8, 22; cycles every 4 weeks.

### Mutational analysis

*KRAS *and *BRAF *genetic analyses were performed on paraffin-embedded tissue blocks from the primary tumor and/or metastatic sites. Genotype status was assessed for *KRAS *codon 12 and 13 mutations and *BRAF *c.1799 T>A (V600E) mutation by SNaPshot^® ^multiplex screening for *KRAS *mutations and *KRAS/BRAF *mutations in 36 and 32 samples, respectively [26,27]; direct sequencing was performed for detection of *KRAS *mutations in 23 samples and to confirm detected mutations. After treatment with xylene thyocyanate and selection of tumoral cell clusters, DNA was isolated using the RecoverAll™ Total Nucleic Acid Isolation Kit for FFPE Tissues (Applied Biosystems, Courtaboeuf, France) according to manufacturer's instructions. When considering the contamination of tumoral samples by non-malignant cells, a *KRAS *mutation in the tumor was defined as appearance of a mutant peak with a height of at least one-third compared to the wild-type.

### SNaPshot and Direct Sequencing assays

SNaPshot multiplex assay was performed as elsewhere reported [26,27]. Briefly, *KRAS *exon 2 and *BRAF *exon 15 were simultaneously amplified by polymerase chain reaction (PCR) using specific primers and purified using NucleoSpin^® ^Extract II kit (Macherey-Nagel EURL, Hoerdt, France). PCR-amplified DNA was analyzed using the ABI PRISM SNaPshot Multiplex kit (Applied Biosystems, Foster City, CA, USA) and five primers including an additional tail at their 5' end allowing their simultaneous detection. Sense primers allowing the extension at nucleotides *KRAS *c.34G, c.35G, c.37G, c.38G and *BRAF *c.1799T were used and a multiplex SNaPshot reaction was performed as reported [26]. *KRAS *exon 2 sequencing was performed from PCR-amplified tumor DNA using the Big Dye V3.1 Terminator Kit (Applied Biosystems, Foster City, CA, USA). Labeled products were separated using an ABI Prism 3130*xl *Genetic Analyzer (Applied Biosystems, Foster City, CA, USA). Data were analyzed using the GeneMapper Analysis Software version 4.0 (Applied Biosystems, Foster City, CA, USA).

### Study design

A retrospective analysis was planned to evaluate prognostic relevance of *KRAS *genotype on clinical outcome of MCRC patients treated with FIr-B/FOx as first-line treatment. Moreover, patients were classified according to involved metastatic sites, L-L and O/MM [6], to evaluate the relevance of metastatic extension in *KRAS *wild-type and mutant MCRC patients. Patients with L-L metastases were evaluated at baseline and every three cycles of treatment by a multidisciplinary team, consisting of a medical oncologist, liver surgeon and radiologist, to dynamically evaluate resectability defined according to resectability categories previously reported [6]. Resection rate was evaluated in the intent-to-treat population enrolled. Liver metastasectomies were defined as R0, if radical surgery, R1, if radioablation was added. Surgery was recommended >4 weeks after BEV discontinuation. Clinical evaluation of response was made by computed tomography (CT) scan; positron emission tomography (PET) was added based on investigators' assessment.

Clinical criteria of activity and efficacy were ORR, PFS and OS. ORR was evaluated according to Response Evaluation Criteria In Solid Tumors (RECIST) criteria [28]; pathologic complete response was defined as absence of residual cancer cells in surgically resected specimens. The overall activity of integrated medical treatment and secondary liver surgery, consisting of the sum of clinical complete responses (cCR) and liver metastasectomies was also evaluated, as previously reported [6]. PFS and OS were evaluated using the Kaplan-Meier method [29]. PFS and PFS from surgery were defined, respectively, as the length of time from the beginning of treatment or the date of liver metastasectomy and disease progression or death (resulting from any cause) or to the last contact; OS as the length of time between the beginning of treatment and death or to last contact. The Log-rank test was used to compare PFS and OS in *KRAS *wild-type versus mutant, L-L versus O/MM, and KRAS wild-type L-L versus O/MM, and *KRAS *mutant L-L versus O/MM MCRC patients [30].

## Results

### Patient demographics

A total of 59 tumoral samples of 64 enrolled MCRC patients (92%) were available: 46 primary tumors and 13 metastases (7 liver, 4 peritoneal carcinomatosis, 1 local recurrence and 1 lung). Demographic and baseline features of patients were representative of the overall phase II study population (Table 1). The number of MCRC patients with *KRAS *wild-type and mutant genotypes was 31 (53%) and 28 (47%), respectively (Table 1); the male/female ratio was 21/10 and 16/12; synchronous metastatic disease, 21 (68%) and 21 (75%) patients. Patients' distribution according to extension of metastatic disease, L-L and O/MM, was, respectively: overall, 25 (42%) and 34 (58%); *KRAS *wild-type, 12 (39%) and 19 (61%); *KRAS *mutant, 13 (46%) and 15 (54%). Table 2 shows *KRAS *mutations detected in 28 patients: codon 12, 24 patients (85.7%), specifically c.35 G>A 15 patients (53.5%), c.35 G>T 7 patients (25%), c.34 G>A and c.35 G>C, 1 patient each; codon 13, 4 patients (14.2%), c.38 G>A 3 patients (10.7%) and c.37_39 dupl, 1 patient. A total of 32 tumoral samples (54%) were also analyzed for *BRAF *and no *BRAF *mutation was detected; 18 out of 31 *KRAS *wild-type MCRC patients were *KRAS *and *BRAF *wild-type; 14 out of 28 *KRAS *mutant MCRC patients were *BRAF *wild-type. EGFR protein expression was positive in 35 patients (59%) and negative in 24 patients (41%): among *KRAS *wild-type patients, positive in 23 patients (74%) and negative in 8 patients (26%); among *KRAS *mutant patients, positive in 13 patients (40%) and negative in 15 patients (60%).

**Table 1:** Patients' features

	*KRAS *wild-type, no. (%)	*KRAS *mutant, no. (%)	Total no. (%)
No. of patients	31 (53)	28 (47)	59
Male/female	21/10	16/12	37/22
Age, years:			
Median	64	65	63
Range	42 to 73	48 to 73	42 to 73
≥65	13 (42)	13 (46)	26 (44)
WHO performance status:			
0	28 (90)	26 (93)	54 (92)
1 to 2	3 (10)	2 (7)	5 (8)
Metastatic disease:			
Metachronous	10 (32)	7 (25)	17 (29)
Synchronous	21 (68)	21 (75)	42 (71)
Primary tumor:			
Colon	14 (45)	20 (71)	34 (58)
Rectum	17 (55)	8 (29)	25 (42)
Sites of metastases:			
Liver	19 (61)	20 (71)	39 (66)
Lung	7 (23)	5 (18)	12 (20)
Lymph nodes	10 (32)	8 (29)	18 (30)
Local	6 (19)	3 (11)	9 (15)
Other	2 (6)	6 (21)	8 (14)
No. of involved sites:			
1	17 (55)	17 (61)	34 (58)
≥2	14 (45)	11 (39)	25 (42)
Single metastatic sites:			
Liver limited	12 (39)	13 (46)	25 (42)
Other than liver	7 (22)	4 (15)	11 (19)
Lung	2 (6)	2 (7)	4 (7)
Lymph nodes	2 (6)	1 (4)	3 (5)
Local	3 (10)	1 (4)	4 (7)
Multiple metastatic sites	12 (39)	11 (39)	23 (39)
Liver metastases:			
Single	8 (26)	3 (11)	11 (19)
Multiple	11 (35)	11 (39)	22 (37)
Previous adjuvant chemotherapy:	6 (19)	2 (7)	8 (14)
FA/5-FU bolus	3 (10)	-	3 (5)
Capecitabine	-	-	-
FOLFOX4	2 (6)	2 (7)	4 (7)
XelOx	1 (3)	-	1 (2)
Previous radiotherapy:	4 (13)	1 (4)	5 (8)
Radiotherapy alone	-	-	-
Radiotherapy + chemotherapy (5-FU continuous infusion)	2 (6)	-	2 (3)
Radiotherapy + chemotherapy (XELOX)	2 (6)	1 (4)	3 (5)

WHO = World Health Organization.

**Table 2:** *KRAS *mutations

Exon	Codon	Hot spot site	Amino acid	No. of patients	%
2	12			**24**	**40.6**
		c.34 G>A	p.Gly12Ser	1	1.6
		c.35 G>A	p.Gly12Asp	15	25.4
		c.35 G>T	p.Gly12Val	7	11.8
		c.35 G>C	p.Gly12Ala	1	1.6
	13			**4**	**6.7**
		c.37_39 dupl	p.Gly13 dupl	1	1.6
		c.37	-	-	-
		c.38 G>A	p.Gly13Asp	3	5

### Activity and efficacy

**Figure 1:**
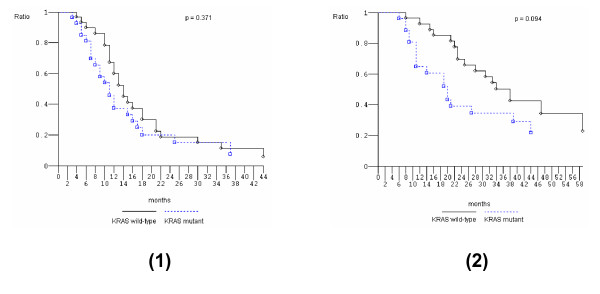
**Kaplan-Meier survival estimate**. Overall population, *KRAS *wild-type versus *KRAS *mutant. 1, Progression-free survival; 2, overall survival.

Overall activity and efficacy data (Table 3) were similar to that reported in the phase II study: ORR was 79% (95% CI 68 to 90); liver metastasectomies were performed in 18 patients (31%), 17 out of 25 L-L patients (68%). After a median follow-up of 21.5 months, median PFS was 12 months (1+ to 69+ months), median OS was 28 months (1+ to 69+ months). Among 30 evaluable *KRAS *wild-type patients, ORR was 90% (95% CI 79 to 100). We observed 27 objective responses: 23 partial responses (77%) and 4 complete responses (CRs) (13%); 2 stable diseases (7%); 1 progressive disease (3%). Disease control rate was 97% (95% CI 90 to 100). Liver metastasectomies were performed in 11 patients (35%), 10 out of 12 L-L patients (83%). Median PFS was 14 months (1+ to 69+ months), 25 events occurred. Median OS was 38 months (1+ to 69+ months), 17 events occurred. Among the 18 *KRAS/BRAF *wild-type patients, ORR was 83% (95% CI 69 to 97), median PFS was 13 months (4 to 44 months), median OS was 31 months (8 to 66+ months). Among 27 evaluable *KRAS *mutant patients, ORR was 67% (95% CI 49 to 85). We observed 18 objective responses: 17 partial responses (63%) and 1 CR (4%); 4 progressive diseases (16%). Disease control rate was 85% (95% CI 71 to 99). Liver metastasectomies were performed in 7 patients (25%) out of 13 L-L patients (54%). Median PFS was 11 months (1+ to 60+ months), 20 events occurred. Median OS was 20 months (1+ to 60+ months), 17 events occurred. Overall, R0 liver resections made up 13 out of 18 liver metastasectomies (72%). Pathologic CRs were obtained in 2 patients (11%), both *KRAS *mutant patients, harboring codon 12 mutations, c.35 G>T and c.34 G>A, with multiple liver-only metastases. In one *KRAS *wild-type patient with single liver associated with lung metastases, double metastatic resections were performed. *KRAS *wild-type compared with mutant patients did not show significantly different PFS nor OS, even if OS seems to be favorable in *KRAS *wild-type patients (Figure 1).

**Table 3:** Activity, efficacy and effectiveness of FIr-B/FOx regimen according to *KRAS *genotype

	Intent to treat analysis					
	
	*KRAS *wild-type		*KRAS *mutant		All	
	
	No	%	No	%	No	%
Enrolled patients	31	100	28	100	59	100
Evaluable patients	30	97	27	96	57	97
Objective response	27	90 (95% CI 79 to 100)	18	67 (95% CI 49 to 85)	45	79 (95% CI 68 to 100)
Partial response	23	77	17	63	40	70
Complete response	4	13	1	4	4	7
Stable disease	2	7	5	18.5	7	12
Progressive disease	1	3	4	15	5	9
Median PFS, months	14		11		12	
Range	1+-69+		1+-60+		1+-69+	
Progression events	25	81	20	71	45	76
Median OS, months	38		20		28	
Range	1+-69+		1+-60+		1+-69+	
Deaths	17	55	17	61	34	58
Liver metastasectomies	11		7		18	
No/overall patients	11/31	35	7/28	25	18/59	31
No/patients with liver metastases	11/19	58	7/20	35	18/39	46
No/patients with L-L metastases	10/12	83	7/13	54	17/25	68
Pathologic complete responses	-	-	2	28.5	2	11

L-L = liver limited; OS = overall survival; PFS = progression-free survival.

**Figure 2:**
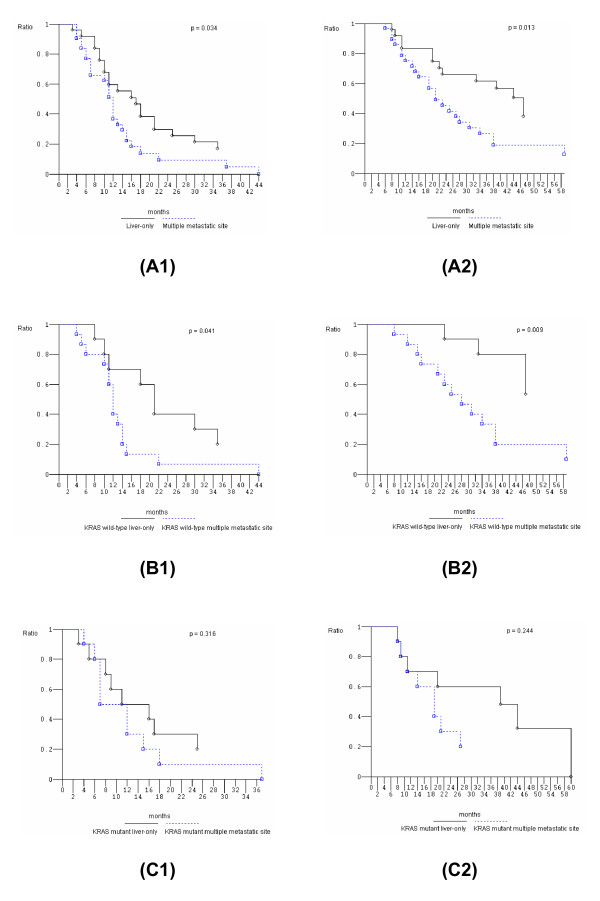
**Overall survival, Kaplan-Meier survival estimate**. **(A) **Liver only versus multiple metastatic sites; **(B) **liver-only versus multiple metastatic sites, *KRAS *wild-type; **(C) **liver-only versus multiple metastatic sites, *KRAS *mutant. 1, Progression-free survival; 2, overall survival.

We verified previously reported findings of significantly different outcome (PFS and OS) with FIr-B/FOx according to extension of metastatic disease [6] in *KRAS *wild-type and mutant patients (Table 4). Among 25 evaluable L-L patients, ORR was 84% (95% CI 69 to 99); overall activity was 80% due to 17 performed liver metastasectomies (68%) and 3 cCRs (12%) in patients who did not undergo liver surgery showing PFS of 69+, 60+, and 40+ months, respectively; median PFS was 17 months (3 to 69+ months); median OS was 47 months (8 to 69+ months). Among the 17 L-L patients who underwent liver metastasectomies, the median PFS was 18 months (8 to 35+ months); median OS was 47 months (10+ to 56+ months). Among 32 evaluable O/MM patients, the ORR was 80% (95% CI 64 to 96), overall activity was 9% due to 1 performed liver plus lung metastasectomies (3%) and 2 cCRs (6%) in patients who did not undergo liver surgery and showing PFS of 22, and 4+ months, respectively; median PFS was 12 months (1+ to 44 months); median OS was 21 months (1+ to 66+ months). Clinical outcome (PFS and OS) in L-L compared to O/MM patients was significantly different (Figure 2A).

**Table 4:** Activity, efficacy and effectiveness of FIr-B/FOx regimen according to *KRAS *genotype and extension of metastatic disease

	All		*KRAS *wild-type		*KRAS *mutant	
	
	L-L	O/MM	L-L	O/MM	L-L	O/MM
Evaluable patients	25	32	12	18	13	14
Objective response (%; 95% CI)	21 (84; 69 to 99)	24 (80; 64 to 96)	12 (100)	15 (80; 59 to 100)	9 (67; 40 to 94)	9 (80; 54 to 100)
Partial response	18	22	10	13	8	9
Complete response	3 (12)	2 (6)	2 (17)	2 (11)	1 (8)	-
Stable disease	2	3	-	2	2	3
Progressive disease	2	3	-	1	2	2
Liver metastasectomies, N (%)	17 (68)	1 (3)	10 (83)	1 (6)	7 (54)	-
Pathologic complete responses	2 (12)	-	-	-	2	-
Overall activity^a^, N (%)	20 (80)	3 (9)	12 (100)	3 (17)	8 (62)	-
Median PFS, months	17	12	21	12	11	11
Range	3-69+	1+44	8-69+	4-44	3-60+	1+37
Progression events	20	27	10	15	10	12
*P *value	0.034		0.044		0.354	
Median OS, months	47	21	47	28	39	19
Range	8-69+	1+66+	18+69+	1+66+	8-60+	1+59+
Deaths	12	23	4	13	8	10
*P *value	0.013		0.017		0.225	

^a^Clinical complete response plus metastasectomies.

L-L = liver limited; O/MM = other/multiple metastatic site; OS = overall survival; PFS = progression-free survival.

Among the 30 evaluable *KRAS *wild-type patients, ORR in 12 L-L and 18 O/MM patients were 100% and 80%, respectively. Overall activity was 100% (ten liver metastasectomies and two cCRs) in L-L and 17% (one liver plus lung metastasectomy and two cCRs) in O/MM patients, respectively. Significantly different clinical outcome was confirmed in L-L compared to O/MM, respectively (Figure 2B): median PFS 21 months (8 to 69+ months) versus 12 months (4 to 44 months) (p 0.044); median OS 47 months (18+ to 69+ months) versus 28 months (1+ to 66+ months) (p 0.017). Among the 27 evaluable *KRAS *mutant patients, ORR in 13 L-L and 14 O/MM patients were 67% and 80%, respectively. Overall activity in L-L patients was 62% (seven liver metastasectomies and one cCR) while no liver metastasectomy nor cCR was obtained in O/MM patients. The comparison of PFS and OS in *KRAS *mutant L-L and O/MM patients was not significantly different: median PFS 11 months (3 to 60+ months) versus 11 months (1+ to 37 months), respectively; median OS 39 months (8 to 60+ months) versus 19 months (1+ to 59+ months), respectively (Figure 2C).

## Discussion

In *KRAS *wild-type patients, BEV addition to doublet chemotherapy significantly increased ORR, PFS and OS up to 60% to 61%, 10.5 to 13.5 months and 21.8 to 27.7 months, respectively [18,21,31,32]. Randomized studies of anti-EGFR added to doublets, in EGFR-overexpressing patients, reported ORR 50% to 61%, PFS 7.7 to 10.6 months, OS 22.4 to 24.9 months [22,23,31-33]. First-line cetuximab plus FOLFOX4, significantly improved ORR and PFS in *KRAS/BRAF *wild-type population, similarly to *KRAS *wild-type patients [34]. In *KRAS *mutant patients, BEV addition to doublet chemotherapy (IFL) significantly increased median PFS up to 9.3 months, while ORR was equivalent to doublet arm (43.2% and 41.2%, respectively), and median OS increased up to 19.9 months, even if not significantly [21,35].

In *KRAS *wild-type and mutant MCRC patients, BEV addition to triplet chemotherapy, according to FIr-B/FOx schedule, reported high activity and efficacy: ORR 90% and 67%, median PFS 14 and 11 months, median OS 38 and 20 months, respectively. A similar clinical outcome was also obtained in *KRAS/BRAF *wild-type patients. Equivalent efficacy was reported with FOLFOXIRI/BEV regimen: ORR 82% and 71%, median PFS 13.6 and 12.6 months, respectively [3]. In unresectable colorectal liver metastases, ORR 79%, median PFS 14 months, median OS 37 months were reported with chrono-IFLO/cetuximab [5].

Median PFS and OS values of MCRC patients treated with FIr-B/FOx were different in *KRAS *wild-type and mutant patients, even if not significantly, while they were equivalent in the FOLFOXIRI plus BEV study [3]. BEV addition to doublet IFL chemotherapy gave median PFS 13.5 and 9.3 months, median OS 27.7 and 19.9 months in *KRAS *wild-type and mutant patients, respectively [18,21]. Significantly better prognosis was reported in *KRAS/BRAF *wild-type patients compared with patients harboring mutations in the *KRAS *or *BRAF *genes (HR 0.51) [18]. Direct comparison of OS between *KRAS *wild-type and mutant MCRC patients treated with BEV-containing chemotherapy failed to significantly differentiate prognosis, as in the present study. Thus, intensive regimens adding BEV to triplet chemotherapy can further increase activity and efficacy in *KRAS *wild-type and mutant patients. Randomized studies would be able to properly evaluate this.

The high activity of triplet chemotherapy plus BEV regimens correlated with increased resection rate of liver metastases and pathologic CR, particularly in L-L MCRC patients [1,3,4,6]. We recently reported that the clinical outcome of L-L compared to multiple metastatic disease was significantly improved up to median PFS 17 months and median OS 44 months [6] due to the effectiveness of integrated FIr-B/FOx intensive treatment and secondary liver surgery. The present analysis confirms the significantly favorable prognosis of L-L compared to MM patients and show that *KRAS *wild-type L-L patients, accounting for 20% of fit MCRC patients, could gain 100% overall activity with an integrated medical and surgical approach, due to performed liver metastasectomies and long-lasting cCRs; median PFS 21 months and OS 47 months. A significantly favorable prognosis was demonstrated in *KRAS *wild-type L-L compared to O/MM patients, even if this represents a retrospective, exploratory analysis in a small cohort of MCRC patients. Using neoadjuvant cetuximab with either FOLFOX6 or FOLFIRI for unresectable colorectal liver metastases, metastasectomies were performed in 38% and 30% patients, respectively [36]. Chrono-IFLO/cetuximab reported a 60% R0 resection rate in unresectable colorectal liver metastases, with ORR 79%, median PFS 14 months and median OS 37 months [5]. Further prospective studies will properly address whether intensive medical treatments, such as FIr-B/FOx, and secondary liver surgery could represent the standard multidisciplinary strategy for *KRAS *wild-type L-L MCRC patients. In *KRAS *mutant patients, prevalently harboring c.35 G>A transversion (53.5%), integrated medical and surgical treatment failed to significantly increase PFS and OS in L-L compared to O/MM patients: median PFS was equivalent (11 months), in spite of 54% performed liver metastasectomies in L-L patients; median OS was 39 and 19 months, respectively. These data should be further evaluated in a larger cohort of MCRC patients. A proper multidisciplinary treatment strategy for *KRAS *mutant patients, showing different aggressiveness [37], sensitivity to medical treatment, and worse clinical behavior, is an unmet need.

## Conclusions

*KRAS *wild-type and mutant genotypes do not significantly affect the clinical outcomes of MCRC patients treated with the first-line FIr-B/FOx intensive regimen. *KRAS *wild-type patients with L-L disease may achieve significantly greater benefit from integration with liver metastasectomies compared to O/MM metastatic extension, with respect to *KRAS *mutant patients. The present findings should be verified in prospective trials of multidisciplinary strategies comparing clinical outcome according to *KRAS *genotype in patients with L-L and O/MM disease.

## Competing interests

The authors declare that they have no competing interests.

## Authors' contributions

Conception and design: GB, ER. Provision of study materials of patients: GB, AD, GC. Collection and/or assembly of data: all authors. Data analysis and interpretation: GB, KC, ER. Manuscript writing: GB, ER. Final approval of manuscript: all authors.

## Pre-publication history

The pre-publication history for this paper can be accessed here:

http://www.biomedcentral.com/1741-7015/10/135/prepub
